# Lack of Innate Interferon Responses during SARS Coronavirus Infection in a Vaccination and Reinfection Ferret Model

**DOI:** 10.1371/journal.pone.0045842

**Published:** 2012-09-24

**Authors:** Mark J. Cameron, Alyson A. Kelvin, Alberto J. Leon, Cheryl M. Cameron, Longsi Ran, Luoling Xu, Yong-Kyu Chu, Ali Danesh, Yuan Fang, Qianjun Li, Austin Anderson, Ronald C. Couch, Stephane G. Paquette, Ndingsa G. Fomukong, Otfried Kistner, Manfred Lauchart, Thomas Rowe, Kevin S. Harrod, Colleen B. Jonsson, David J. Kelvin

**Affiliations:** 1 Division of Experimental Therapeutics, Toronto General Hospital Research Institute, University Health Network, Toronto, Ontario, Canada; 2 University of Toronto, Toronto, Ontario, Canada; 3 Institute of Medical Science, Faculty of Medicine, University of Toronto, Toronto, Ontario, Canada; 4 Department of Immunology, Faculty of Medicine, University of Toronto, Toronto, Ontario, Canada; 5 International Institute of Infection and Immunity, Shantou University Medical College, Shantou, Guangdong, China; 6 Department of Biochemistry and Molecular Biology, Southern Research Institute, Birmingham, Alabama, United States of America; 7 Lovelace Respiratory Research Institute, Albuquerque, New Mexico, United States of America; 8 Baxter Innovations GmbH, Vienna, Austria; 9 Center for Predictive Medicine, Louisville, Kentucky, United States of America; 10 Immune Diagnostics & Research, Toronto, Ontario, Canada; 11 Sezione di Microbiologia Sperimentale e Clinica, Dipartimento di Scienze Biomediche, Università degli Studi di Sassari, Sassari, Italy; Kantonal Hospital St. Gallen, Switzerland

## Abstract

In terms of its highly pathogenic nature, there remains a significant need to further define the immune pathology of SARS-coronavirus (SARS-CoV) infection, as well as identify correlates of immunity to help develop vaccines for severe coronaviral infections. Here we use a SARS-CoV infection-reinfection ferret model and a functional genomics approach to gain insight into SARS immunopathogenesis and to identify correlates of immune protection during SARS-CoV-challenge in ferrets previously infected with SARS-CoV or immunized with a SARS virus vaccine. We identified gene expression signatures in the lungs of ferrets associated with primary immune responses to SARS-CoV infection and in ferrets that received an identical second inoculum. Acute SARS-CoV infection prompted coordinated innate immune responses that were dominated by antiviral IFN response gene (IRG) expression. Reinfected ferrets, however, lacked the integrated expression of IRGs that was prevalent during acute infection. The expression of specific IRGs was also absent upon challenge in ferrets immunized with an inactivated, Al(OH)_3_-adjuvanted whole virus SARS vaccine candidate that protected them against SARS-CoV infection in the lungs. Lack of IFN-mediated immune enhancement in infected ferrets that were previously inoculated with, or vaccinated against, SARS-CoV revealed 9 IRG correlates of protective immunity. This data provides insight into the molecular pathogenesis of SARS-CoV and SARS-like-CoV infections and is an important resource for the development of CoV antiviral therapeutics and vaccines.

## Introduction

Severe Acute Respiratory Syndrome (SARS) disease hit the world in late 2002 and in 4 months swiftly spread to 29 countries infecting over 8,000 people and killing over 700 [1]. The etiological agent of SARS disease was determined to be of the coronavirus (CoV) family; the largest family of single-stranded, positive-sense RNA genomes known [1]. The overall mortality rate of SARS corona virus (SARS-CoV) infection was ∼10% but this rate was 50% in patients over 65. Prior to the emergence of the SARS virus, coronaviruses were known to cause mild upper-respiratory tract diseases in humans. In contrast, SARS-CoV infection caused severe disease in the lower respiratory tract disease with symptoms ranging from flu-like and viral pneumonia to acute respiratory distress syndrome (ARDS) and fatal outcome [2]–[5]. The virus emerged from the Guangdong Province in China where it crossed to humans from a zoonotic reservoir. The most established theory puts horseshoe bats as the ultimate reservoir for the SARS-CoV and implicates palm civets as the intermediate species that passed the virus to humans [1]. Aggressive public health intervention strategies are credited with successfully minimizing the SARS-CoV infection range, although it is uncertain if these same public health strategies would sufficiently contain a future SARS-CoV or SARS-like-CoV outbreak due to virus evolution.

Importantly, coronaviruses have a propensity toward frequent host-shifting events and over the past 30 years there have been many CoV cross-species transmission incidents giving rise to new animal and human CoV -based diseases. Coronaviruses infect a broad range of species lending further chance for recombination events and the advent of new CoV species. Moreover, coronaviruses can change cell type, tissue and host species barriers with ease [6], [7]. Typically, the spike (S) protein of coronaviruses determines the host infectivity and the organization of the SARS-CoV S protein shows significant similarity with other aggressive class I viral fusion proteins: influenza virus HA, HIV-1 Env, Simian virus 5, and Ebola virus Gp2 [1]. The promiscuity of coronaviruses coupled with the tendency for mutations to occur gives reason for concern that another CoV outbreak is likely and highlights the need for continuous viral surveillance and forward development of CoV vaccination strategies and therapeutics.

Although entry of SARS-CoV into mammalian cells has been determined to be facilitated by the angiotensin-1 converting enzyme 2 (ACE2) molecule [8], the mechanisms by which the virus evades host immune responses causing generalized inflammation, increasing viral burden, and severe lung pathology still remain a significant scientific problem. Previous studies have shown substantial problems with potential CoV vaccines where the vaccines cause disease exacerbation opposed to initiating immunological protection [9], [10]. Recently, several groups have described the immunologic response during SARS-CoV infection [11] and some have investigated the use of a mouse adapted SARS-CoV in the mouse model [12]–[15]. The mouse-adapted SARS-CoV (MA15) is a valuable animal model for investigating the immune response and possible therapeutic and prophylactic strategies for SARS-CoV disease. Although the model helped to elucidate immune-pathological events during SARS-CoV infection and protection [12]–[15], the caveat of this model is that it is based on an adapted virus and not a wild-type SARS-CoV that has naturally occurred in nature and cause disease in humans and animals. Although death is not observed in our wt TOR2 SARS-CoV ferret model, there are still several advantages where the use of both models is perhaps of equal importance as results from the mouse model compliment findings from the ferret model and vice versa. Specifically, use of the ferret model provides several benefits. As mentioned above, ferrets are susceptible to wild-type SARS-CoV infection from strains isolated from humans [16], [17]. Furthermore, when infected with respiratory viruses including the SARS-CoV ferrets display many of the symptoms and pathological features as seen in infected humans as ferrets and humans have similar lung physiology [18]–[21]. Quantitative clinical signs displayed by ferrets include a rise in core body temperature (fever), nasal discharge (sneezing and runny nose) and weight loss [16].

Here we investigated the immune response transcriptome of SARS-CoV pathogenesis in a ferret model infected with an unadapted SARS-CoV and subsequently evaluated gene expression signatures induced with SARS-CoV reinfection. Furthermore, ferrets were immunized with a SARS-CoV vaccine and then challenged to compare immunological profiles with the SARS-CoV reinfected animals. The objective of this study was to identify immune correlates of protection upon reinfection with SARS-CoV in ferrets and provide a comprehensive profile of an effective and nonpathological immune response to SARS-CoV challenge following immunization. This information will not only provide a foundation for direct comparison with future SARS vaccine studies, but will also allow us to determine what immune mediators are responsible for the successful antiviral response.

## Results

### Effective Immune Responses to SARS-CoV Reinfection

Previously, the lack of a representative SARS-CoV infection animal model has limited the ability to uncover immunopathogenic mechanisms of SARS and has impeded progress in vaccination strategies. Currently, a mouse model for SARS-CoV infection has described aspects of SARS-CoV pathogenesis although these studies utilized a mouse adapted SARS virus [12], [13], [15]. The ferret, *Mustela putorius furo*, displays many of the symptoms and pathological features seen in SARS-CoV infected humans and is susceptible to unadatped SARS-CoV strains therefore suggesting it as a useful animal model for the study of SARS-CoV infection and vaccination strategies [5], [22]–[24]. Here ferrets were assigned to three treatment groups for this study: (A) a mock infection control group, (B) a single infection group, and (C) a group that was infected at the same time as group B, then subsequently reinfected 29 days later. The infected groups were innoculated intranasally (IN) with 10^3^ TCID_50_ (50% tissue culture infective dose) of SARS-CoV TOR2 strain, while the mock infected animals received intranasal instillation of vehicle (serum-free cell culture medium (SFM)).

Ferrets were monitored for clinical signs of disease twice daily. The most significant symptom observed in SARS-CoV infected animals was sneezing, which was observed in one third of the infected ferrets between 4 and 11 days post-infection (DPI). Sneezing was not observed in ferrets from the control group (A), nor in group (C) at any time post- reinfection.

**Figure 1:**
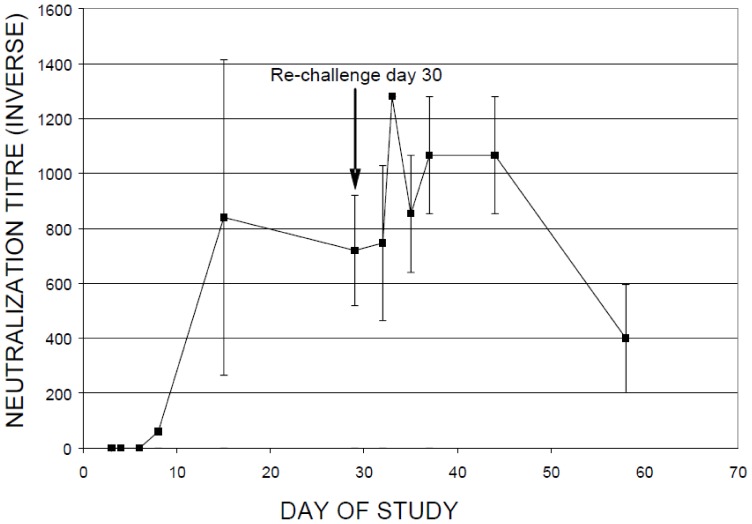
Mean levels of serum neutralizing antibody to SARS-CoV. Neutralizing antibody levels in serum were determined as outlined in the materials and methods section. Inverse neutralization titre is represented on the y-axis vs. day of study on the x-axis. Note that ferrets were infected on study day 1 and reinfected ferrets were also innoculated on study day 30. Mock infected animals received an intranasal instillation of serum-free media on study day 1 and had undetectable titres. Values shown represent group mean of 3–4 ferrets per group, and error bars show standard deviation.

**Figure 2:**
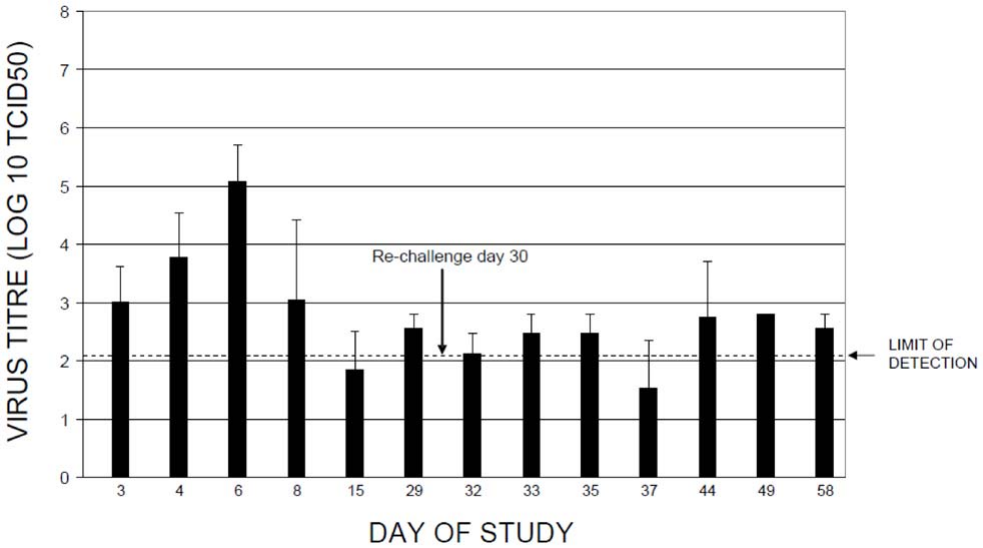
Viral burden in lung tissue. Viral burdens in sections of lung were determined by the TCID_50_ method, as outlined in the materials and methods section. Log10 virus titres are shown on the vertical axis vs. study day on the horizontal axis. Note that ferrets were infected on study day 1, and reinfected ferrets were also inoculated on study day 30. Mock infected animals received an intranasal instillation of serum-free media on study day 1 and had undetectable virus. Values shown represent group means of 3–4 ferrets per group, and error bars show standard deviation.

**Figure 3:**
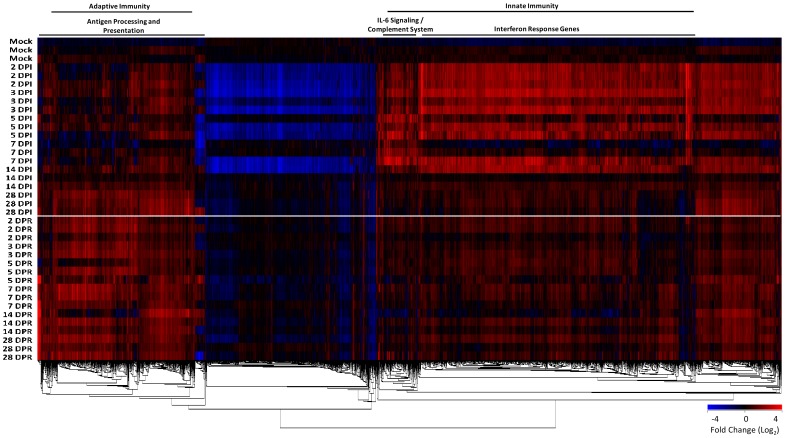
Lung histopathology to SARS-CoV challenge following reinfection. Histological lung sections (5 µm) were obtained from multiple lung lobes at 7 d postchallenge and stained by hematoxylin and eosin. Representative micrographs from uninfected (A), SARS infected alone (B), or SARS infected and re-infected (C and D) are shown. Primary SARS-CoV infection produced inflammation and the appearance of lung immune cells primarily surrounding small-to-medium bronchial airways at 7 days following challenge (B). Ferrets that received infection-reinfection were largely protected from lung histopathology (C).

Neutralising antibody titers rose above baseline from 6 DPI to maximal post-infection levels at 15 DPI (**Figure 1**). Specifically, neutralizing antibodies in the single infection group (B) increased from study Day 8 to a peak titer (820U) on Day 15. Neutralizing antibody titers fell slightly to 720U on Day 30 (day of reinfection). Increases in neutralizing antibodies directly correlated with decreased viral burden in the lung (**Figure 2**). At four weeks post-infection, animals in group C were reinfected with SARS-CoV and a second spike in neutralizing antibodies (1300U) above levels seen during the infection phase was observed within a week. Neutralizing antibody titers remained elevated for approximately two weeks, then diminished to 400U by the end of the study (day 58). The boost in antibody titer may have contributed to the lower viral burden observed during the reinfection phase (Days 32 to 58). This second peak in neutralizing antibody titres was coincident with restricted viral replication (**Figure 2**). Neutralizing antibodies were not detected in the mock-infection control group. The TOR2 strain of SARS-CoV replicated well in the upper respiratory tract (data not shown) and lung within the first week post-infection. Significant viral titers above background were detected in lung, ranging from one to three log above background. Peak viral titers were observed between 3 to 6 DPI in the lung (**Figure 2**). Viral titers post reinfection (Days 30 to 58) were generally restricted below 2 log values (**Figure 2**). Furthermore we investigated the lung histopathology following SARS-CoV infection and following SARS-CoV infection-reinfection (**Figure 3**). Ferret lung sections were obtained from multiple lung lobes at 7 d post primary infection or 7 d post secondary infection and stained by hematoxylin and eosin. Primary SARS-CoV infection produced inflammation and the appearance of lung immune cells primarily surrounding small-to-medium bronchial airways at 7 days following challenge (**Figure 3** **B**). Ferrets that received infection-reinfection were largely protected from lung histopathology (**Figure 3** **C**). Taken together the ferret mounted an effective immune response to the reinfection with SARS-CoV, with increased neutralizing antibody titre, restricted virus replication, diminished clinical symptoms and less lung pathology in comparison to the initial infection of the naive host.

### Lack of IRG Expression in SARS-CoV-reinfected Ferrets

**Figure 4:**
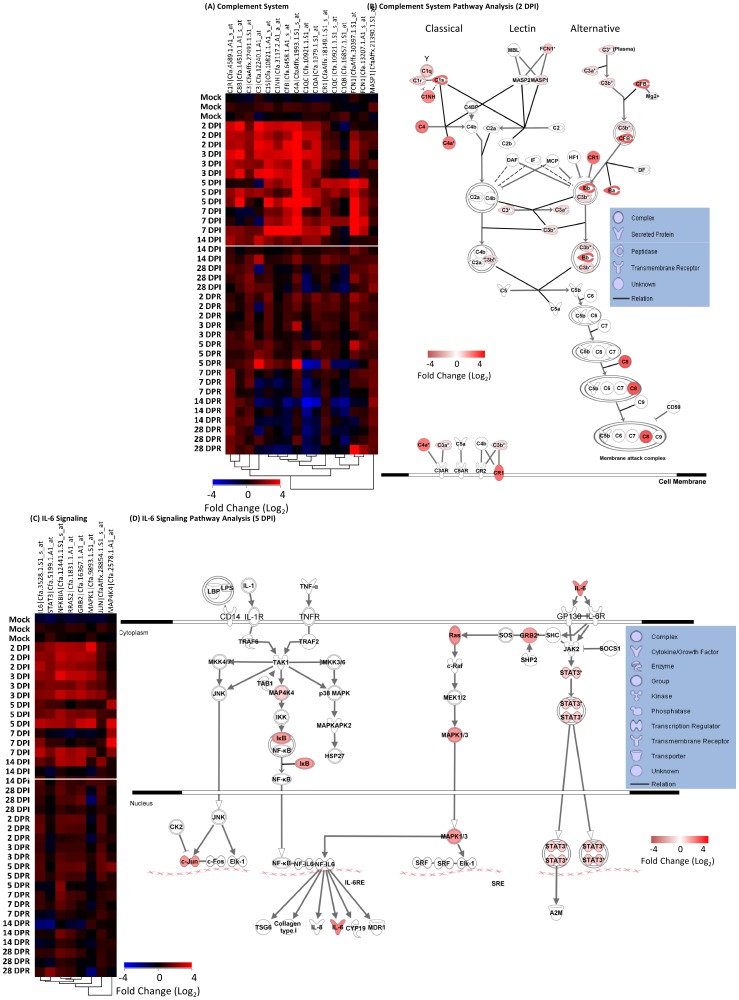
Microarray analysis of gene expression in lung tissue from ferrets infected and reinfected with SARS-CoV. EDGE analysis across all time points identified 3454 genes as significantly differently expressed (≥2-fold change in at least one time point, p≤0.05, and q≤0.1) as described in the Methods. Genes were then one-way hierarchically clustered by gene using Pearson correlation and average distance metrics (red, upregulated; blue, downregulated). The most significant canonical signaling pathways according to IPA for the resulting clusters are noted. Full gene lists are publically available on GEO (see Methods). DPI, days post-infection. DPR, days post-reinfection.

To characterize host immune responses in an infection/reinfection ferret model of SARS-CoV pathogenesis, we performed gene expression profiling on lung tissue taken at necropsy at days 2, 3, 5, 7, 14 and 28 days post-infection (DPI) and days 2, 5, 7, 14 and 28 days post-reinfection (DPR). Three ferrets at each time point were profiled and gene expression signatures associated with pulmonary immunopathology during SARS were identified using an Extraction and Analysis of Differential Gene Expression (EDGE) differential time course microarray analysis [25] on the SARS-CoV infected ferret lung samples. A heat map overview containing 3454 genes identified as significantly differentially expressed across all time points and classified by Ingenuity Pathway Analysis (IPA) into three clusters of functionally related genes (**Figure 4**); an IL-6/complement, an IRG, and an adaptive immune gene cluster. The two innate immune gene clusters, IL-6 signaling/complement and IRG cluster, were strongly expressed in ferret lung tissue from 2–14 DPI with SARS-CoV relative to the mock primary infection group. Conversely, an adaptive immune response gene cluster enriched with genes involved in antigen processing and presentation was highly expressed in the lungs of SARS-CoV infected ferrets after 14 DPI. SARS-CoV titres increased in the lungs prior to the initial peak in neutralizing antibody titers at 14 DPI and then bordered on the limit of detection (**Figure 2**). Upon reinfection with SARS-CoV, the ferrets at 29 DPI did not increase gene expression despite an anamnestic response in neutralizing antibodies at 3 DPR (**Figure 1**). We analyzed the three clusters of functionally related innate immune genes in more detail below.

### Differential Evolution of Gene Expression in Ferret Lungs during SARS-CoV Infection and Reinfection

Our above analysis of the gene expression profiles during SARS-CoV infection and reinfection showed a decrease in IRG expression during SARS-CoV-reinfection compared to primary infection. We next went on to further analyse the expression profiles of SARS infection compared to reinfection using the DAVID bioinformatic tool [26] was used to perform functional classification in differentially expressed genes. During SARS-CoV primary ferret infection, a robust increase in the number of up- and down-regulated genes on day 2 after infection occurred. By day 5 the differential gene expression had decreased markedly although it is still moderate levels and on day 7, the gene expression was found to be close to baseline. During reinfection, a moderate increase in gene expression occurred which was maintained for a longer period of time (**Table 1**). Next, we analyzed the pathway activation profiles on 2 DPI compared to 2 DPR, which corresponded to peak immune activity during both infections (**Table 2**). On 2 DPI, the gene expression patterns showed strong activation of inflammation-related genes, activation of MAPK, JAK-STAT and Wnt signaling pathways (**Table 2**). Together, these pathways participate in leukocyte activation and migration to infection sites. Interestingly, among the upregulated genes on 2DPR, lysosomal degradation was the only among the KEGG pathways to be significantly enriched (Fisher’s exact test’s p = 1.6E−8); this scenario is suggestive of the presence of relevant phagocytosis-mediated immunity but without triggering a significant inflammatory response. Taken together, these results suggest that there are differential gene expression profiles corresponding to SARS infection compared to reinfection that may reflect protective host immune responses.

**Table 1:** Number of regulated genes in different functional categories following primary infection and reinfection.

		Days post primary infection			Days post reinfection		
		2	5	7	2	5	7
Total	↑	3025	1091	231	1151	819	1773
	↓	1348	449	107	228	534	473
Cellular process	↑	1779	668	144	646	444	1028
	↓	683	245	57	117	281	255
Metabolic process	↑	1296	488	95	469	307	754
	↓	489	175	47	83	204	181
Response to stimulus	↑	483	179	66	173	128	273
	↓	221	85	17	44	93	84

Number of regulated genes in different functional categories with at least 1.5-fold change and a significant t-test of p<0.05 (↑ upregulated, ↓ downregulated).

**Table 2:** Intersect analysis of upregulated genes in the lungs of ferrets after infection or reinfection with SARS-CoV.

	2 DPI only	2 DPI & 2 DPR	2 DPR only
Total upregulated genes	2626	399	752
***Gene Ontology***			
Signal transduction	321	41	85
Immune system process	121	11	26
Ubiquitin-dependent protein catabolic process	64	6	16
G-protein coupled receptor protein signalling pathway	63	4	17
Protein kinase cascade	53	5	11
Inflammatory response	45	6	10
***KEGG Pathways***			
MAPK signaling pathway	44	5	7
Wnt signaling pathway	26	4	3
Cytokine-cytokine receptor interaction	24	0	4
Jak-STAT signaling pathway	19	3	4
Cell adhesion molecules (CAMs)	18	3	4
Lysosome	14	9	18
Toll-like receptor signaling pathway	13	2	1

“2DPI only”: specific of 2 days after infection, “2DPR only”: specific of 2 days after reinfection, and “2 DPI & 2DPR”: genes upregulated in both experimental groups. CoV (≥1.5-fold change and Student’s t-test p<0.05).

### Primary but not Secondary SARS-CoV-infection Increases Complement and IL-6 signaling Genes

**Figure 5:**
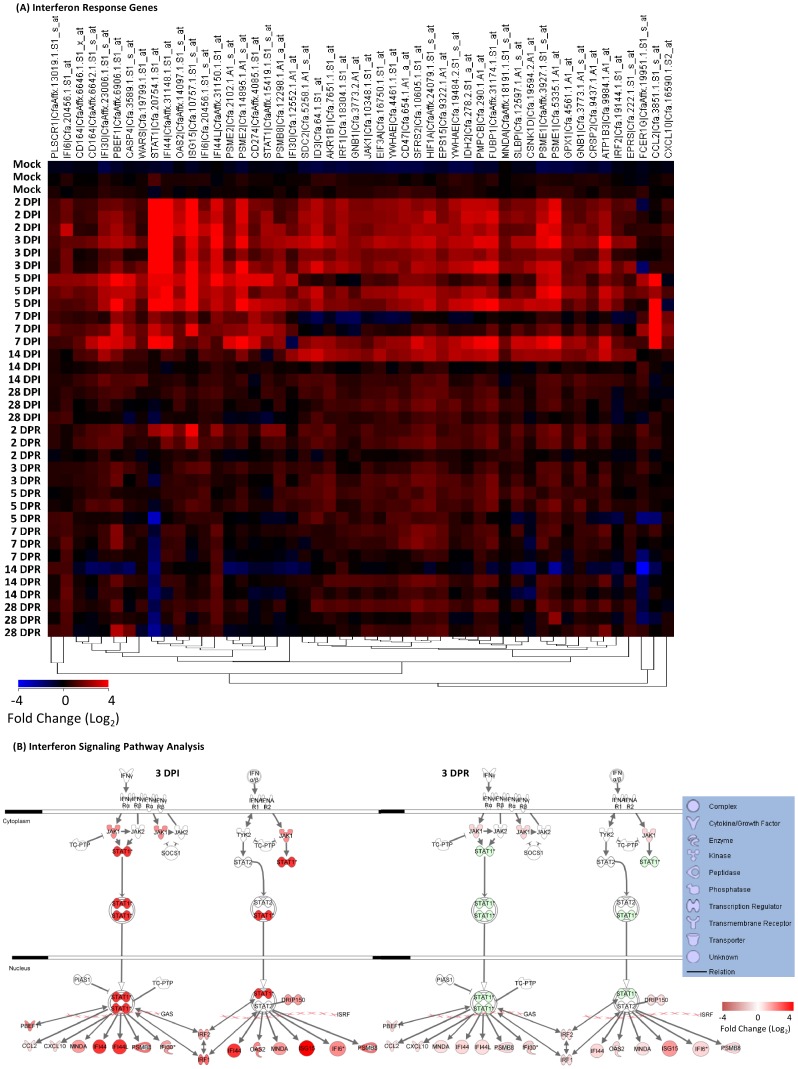
Complement and IL-6 signaling in SARS-CoV infected-reinfected ferret lungs. (A) Complement and IL-6 signaling (C) genes selected by pathway analysis are shown in a one-way hierarchical cluster (red = upregulated, blue = downregulated). (B) IPA canonical complement system pathway analysis at 2 DPI. (D) IPA canonical IL-6 signaling pathway analysis at 5 DPI. All genes are significantly differently expressed (EDGE analysis: ≥2-fold change in at least one time point, p≤0.05, and q≤0.1).

The complement cascade plays an integral part in innate immunity by labeling pathogens for destruction, inducing leukocyte migration and lysing bacterial [27]. We found that a cluster enriched in complement system genes, including C1NH, C1QA/B/C, C1R/S, C3, C4A, C8B, CFB, CR1, FCN1 and MASP1 (see **Table 3** for full gene names), was significantly upregulated during the first 2 weeks post infection (**Figure 5A**). Quantitative RT-PCR (QRT-PCR) validation of C1NH and FCN1 is shown in **Figure S1**. Interestingly, the complement gene cluster was not as significantly upregulated the first 2 weeks post-reinfection and was subsequently markedly downregulated following 7 DPR. The genes of all three complement cascades were integrated at 2 DPI determined by Ingenuity Pathway Analysis (IPA), including the regulators C1NH and CR1 (**Figure 5B**). In summary, complement activation was correlated to the transition from innate to specific immune responses and clearance of SARS-CoV during primary infection and was not strongly regulated during reinfection.

**Table 3:** Expression of selected significantly changed genes over time in SARS-CoV infected–reinfected ferret lungs.

				Gene Expression^a^												
Gene		Gene Name	Ferret-likeGene ID^c^	Days Post Infection						Days Post Reinfection						EDGE p value^b^
				2	3	5	7	14	28	2	3	5	7	14	28	
**Complement System**																
C1NH		C1 inhibitor	Cfa.3117	1.31	1.74	0.97	1.41	0.25	−0.11	−0.03	−0.02	0.39	−0.54	−0.42	−0.51	≤0.001
C1QA		complement component 1, q subcomponent, A chain	Cfa.1379	1.57	1.58	1.28	0.79	0.54	−0.10	0.73	0.20	−0.09	−0.33	−0.98	−0.63	≤0.001
C1QB		complement component 1, q subcomponent, B chain	Cfa.16857	−0.47	−0.20	0.77	0.18	0.16	−0.78	0.19	−0.20	−0.39	−0.47	−1.00	−0.65	0.026
C1QC		complement component 1, q subcomponent, C chain	Cfa.10921	1.32	1.47	1.72	1.01	0.53	−0.82	0.15	−0.42	−0.53	−1.04	−1.60	−1.03	≤0.001
C1QC		complement component 1, q subcomponent, C chain	Cfa.10921	0.18	0.31	1.10	0.55	0.39	−0.22	0.58	0.28	−0.08	−0.04	−0.59	−0.32	0.019
C1R		complement component 1, r subcomponent	Cfa.4589	0.64	0.54	1.13	1.41	0.56	0.34	0.48	0.42	0.63	0.38	0.39	0.27	≤0.001
C1S		complement component 1, s subcomponent	Cfa.10821	1.72	1.93	1.53	1.48	0.37	0.49	0.75	0.59	0.91	−0.05	−0.42	−0.18	≤0.001
C3		complement component 3	Cfa.12240	2.26	1.63	0.14	0.00	1.13	−0.05	−0.32	−0.01	1.72	−0.52	−0.56	−0.32	0.015
C3		complement component 3	CfaAffx.27491	1.03	0.98	0.57	0.10	0.57	0.92	0.64	0.73	0.68	0.54	0.58	0.32	0.044
C4A		complement C4 precursor	CfaAffx.1993	2.35	2.38	2.80	2.25	0.68	0.51	0.67	0.91	1.11	0.52	−0.11	0.21	≤0.001
C8B		complement component 8, beta	Cfa.14510	1.87	1.89	1.43	0.01	0.28	0.54	−0.03	0.26	0.39	0.11	0.51	0.48	≤0.001
CFB		complement factor B	Cfa.6458	1.81	1.66	1.88	2.11	0.50	−0.24	0.42	0.28	0.22	−0.26	−0.93	−0.69	≤0.001
CR1		complement component (3b/4b) receptor 1	CfaAffx.18149	1.61	1.67	0.76	0.39	0.71	0.31	0.25	0.25	0.37	1.12	0.98	1.01	≤0.001
FCN1		ficolin 1	CfaAffx.30397	0.42	0.42	1.36	1.59	0.39	0.55	0.60	0.54	0.38	0.36	0.06	0.54	0.002
FCN1		ficolin 1	Cfa.13207	0.92	0.49	2.61	2.95	0.51	0.66	0.99	0.42	0.61	0.29	−0.28	0.95	0.003
MASP1		mannan-binding lectin serine peptidase 1	CfaAffx.21390	0.35	0.24	0.34	0.17	0.30	1.02	0.34	0.64	0.55	0.84	0.98	0.69	≤0.001
**IL-6 Signaling**																
GRB2		growth factor receptor-bound protein 2	Cfa.16367	1.58	1.50	0.83	0.29	0.67	0.49	0.69	0.60	0.52	0.50	0.22	0.52	0.034
IL6		interleukin 6	Cfa.3528	0.17	0.35	1.44	1.71	0.28	0.16	0.17	0.19	0.61	0.28	0.19	0.12	≤0.001
JUN		jun oncogene	CfaAffx.28854	1.68	1.33	1.20	0.35	0.19	−0.37	−0.30	0.18	−0.07	−0.07	0.05	−0.41	≤0.001
MAP4K4		mitogen-activated protein kinase kinase kinase kinase 4	Cfa.2578	0.67	1.00	0.48	0.20	0.79	0.60	0.69	0.84	0.64	0.45	0.36	0.42	0.033
MAPK1		mitogen-activated protein kinase 1	Cfa.9893	1.53	1.68	1.12	0.60	0.66	0.71	0.67	0.59	0.54	0.24	−0.15	0.11	0.003
NFKBIA		nuclear factor of kappa light polypeptide gene enhancer in B-cells inhibitor, alpha	Cfa.12441	1.31	1.28	1.11	0.46	0.01	0.08	−0.02	−0.04	0.02	−0.07	−0.41	0.43	≤0.001
RRAS2		related RAS viral (r-ras) oncogene homolog 2	Cfa.1831	1.77	1.75	1.23	0.65	0.91	0.70	0.67	0.66	1.01	0.80	0.50	0.69	0.032
STAT3		signal transducer and activator of transcription 3	Cfa.5199	1.38	1.24	1.21	1.14	0.57	0.46	0.79	0.61	0.44	0.28	−0.46	0.28	≤0.001
**Interferon Signaling**																
AKR1B1	aldo-keto reductase family 1, member B1		Cfa.7651	1.23	1.44	0.89	0.48	0.84	0.62	0.66	0.82	0.77	0.33	0.33	0.38	0.039
ATP1B3	ATPase, Na+/K+ transporting, beta 3		Cfa.9984	1.44	1.98	1.60	1.04	0.88	0.37	0.53	0.68	0.26	0.55	0.24	0.72	0.005
CASP4	caspase 4		Cfa.3589	0.91	0.82	1.86	1.36	0.54	0.00	0.35	0.29	0.12	0.28	−0.19	0.40	≤0.001
CCL2	chemokine (C-C motif) ligand 2		Cfa.3851	0.19	0.24	3.66	4.49	−0.01	0.07	0.24	0.12	−0.26	0.19	−0.23	−0.18	≤0.001
CD164	CD164 molecule, sialomucin		CfaAffx.6642	0.51	0.54	1.18	1.07	0.57	0.22	0.53	0.47	0.46	0.00	−0.37	−0.04	≤0.001
CD164	CD164 molecule, sialomucin		CfaAffx.6646	0.47	0.43	1.03	0.66	0.42	0.09	0.35	0.37	0.28	0.17	0.00	0.03	0.003
CD274	CD274 molecule		CfaAffx.4085	0.98	1.05	1.66	1.78	0.16	−0.01	0.14	0.17	0.11	0.00	−0.30	−0.15	≤0.001
CD47	CD47 molecule		Cfa.654	1.51	1.31	1.52	0.44	0.74	0.54	0.56	0.58	0.74	0.66	0.55	0.44	0.038
CRSP2	Cofactor for Sp1 transcriptional activation subunit 2		Cfa.9437	1.06	1.02	0.72	0.48	0.67	0.14	0.34	0.16	0.14	0.41	−0.07	0.39	0.023
CSNK1D	casein kinase 1, delta		Cfa.19594	1.11	1.44	1.13	0.84	0.56	0.04	0.26	0.16	0.22	−0.36	−0.72	−0.12	≤0.001
CXCL10	chemokine (C-X-C motif) ligand 10		Cfa.16590	0.60	0.39	−0.01	1.11	0.11	0.05	0.10	0.01	0.12	0.07	0.00	0.09	0.044
EIF3A	eukaryotic translation initiation factor 3, subunit A		Cfa.16750	1.33	1.43	1.01	0.37	0.87	0.60	0.41	0.49	0.48	0.43	0.25	0.46	0.016
EPRS	glutamyl-prolyl-tRNA synthetase		Cfa.222	0.63	1.06	0.57	0.08	0.48	0.10	0.06	0.24	0.14	0.43	0.25	0.58	0.038
EPS15	epidermal growth factor receptor pathway substrate 15		Cfa.9322	1.29	1.34	1.18	0.70	0.94	0.78	0.50	0.71	0.64	0.76	0.50	0.79	0.028
FCER1G	Fc fragment of IgE, high affinity I, receptor for; gamma		CfaAffx.19951	0.03	−0.22	0.97	0.90	0.42	0.06	0.60	0.47	−0.15	−0.30	−1.32	−0.35	0.011
FUBP1	far upstream element binding protein 1		CfaAffx.31174	1.95	2.07	1.70	1.20	0.87	0.85	0.81	0.94	0.75	0.66	0.13	0.63	0.006
GNB1	guanine nucleotide binding protein, beta 1		Cfa.3773	0.95	1.03	1.23	0.94	0.71	0.50	0.68	0.81	0.61	0.49	0.33	0.60	0.031
GNB1	guanine nucleotide binding protein, beta 1		Cfa.3773	1.20	1.32	0.54	−0.08	0.55	0.42	0.34	0.20	0.41	0.40	0.33	0.26	0.029
GPX1	glutathione peroxidase 1		Cfa.4561	0.96	1.07	1.08	0.57	0.65	0.24	0.23	0.28	0.44	−0.05	−0.03	0.18	0.004
HIF1A	hypoxia-inducible factor 1, alpha subunit		CfaAffx.24079	1.66	1.72	1.65	0.90	1.02	0.95	0.85	0.96	0.89	0.90	0.37	0.70	0.012
ID3	inhibitor of DNA binding 3		Cfa.64	1.76	1.86	1.32	0.17	1.05	0.55	0.43	0.63	0.80	0.19	0.27	0.36	0.019
IDH2	isocitrate dehydrogenase 2		Cfa.278	1.88	1.72	1.41	0.70	0.63	0.46	0.44	0.34	0.43	0.30	0.15	0.16	0.002
IFI30	interferon, gamma-inducible protein 30		CfaAffx.23006	1.15	1.05	1.75	1.20	1.00	0.40	0.74	0.61	0.47	0.44	0.03	0.16	0.004
IFI30	interferon, gamma-inducible protein 30		Cfa.12552	0.85	1.01	1.34	0.07	0.69	0.01	0.10	0.23	0.31	0.06	−0.15	−0.07	0.035
IFI44	interferon-induced protein 44		CfaAffx.31148	4.21	3.22	3.08	1.61	0.25	0.23	1.02	0.32	0.22	0.54	0.09	0.02	≤0.001
IFI44L	interferon-induced protein 44-like		CfaAffx.31150	2.17	2.30	2.11	0.99	0.56	0.49	0.76	0.28	0.24	0.77	0.81	0.77	≤0.001
IFI6	interferon, alpha-inducible protein 6		Cfa.20456	1.32	1.04	1.08	0.60	0.11	0.55	0.12	0.31	0.27	0.74	0.82	0.75	≤0.001
IFI6	interferon, alpha-inducible protein 6		Cfa.20456	1.45	1.36	1.53	0.85	0.48	0.48	0.58	0.73	0.63	0.31	0.20	0.45	≤0.001
IRF1	interferon regulatory factor 1		Cfa.18304	1.59	1.56	0.83	0.03	0.60	0.50	0.30	0.23	0.47	0.40	0.60	0.45	0.007
IRF2	interferon regulatory factor 2		Cfa.19144	0.73	0.91	1.11	0.29	0.65	−0.36	0.06	0.10	−0.11	−0.09	−0.43	0.03	0.002
ISG15	ISG15 ubiquitin-like modifier		Cfa.10757	4.12	2.49	3.77	1.42	0.09	0.24	1.40	0.63	0.58	−0.07	0.09	0.16	≤0.001
JAK1	Janus kinase 1		Cfa.10348	1.16	1.19	1.03	0.31	0.66	0.58	0.51	0.66	0.65	0.48	0.35	0.59	0.039
MNDA	myeloid cell nuclear differentiation antigen		CfaAffx.18191	0.96	1.03	0.76	0.46	0.13	0.29	0.40	0.25	0.18	0.06	−0.08	−0.05	0.002
OAS2	2′-5′-oligoadenylate synthetase 2		CfaAffx.14097	1.90	1.28	1.77	0.83	0.35	0.47	0.73	0.54	0.40	0.54	0.30	0.55	≤0.001
PBEF1	pre-B-cell colony enhancing factor 1		CfaAffx.6906	1.34	1.28	2.43	1.89	0.77	0.35	0.65	0.97	0.46	0.96	0.26	1.02	≤0.001
PLSCR1	phospholipid scramblase 1		CfaAffx.13019	0.65	0.52	1.02	0.71	0.28	0.34	0.21	0.32	0.24	0.28	0.30	0.36	0.002
PMPCB	peptidase (mitochondrial processing) beta		Cfa.290	1.78	1.93	1.62	0.92	0.82	0.68	0.62	0.68	0.73	0.63	0.61	0.62	0.015
PSMB8	proteasome (prosome,macropain) subunit, beta, 8		Cfa.12298	0.89	1.48	1.50	1.22	0.33	0.28	0.71	0.69	0.28	0.02	−0.31	0.01	≤0.001
PSME1	proteasome (prosome,macropain) activator subunit 1		CfaAffx.3927	1.91	1.86	1.95	1.24	0.85	0.52	0.62	0.77	0.57	0.59	0.43	0.59	≤0.001
PSME1	proteasome (prosome,macropain) activator subunit 1		Cfa.5335	2.23	2.01	2.20	1.37	0.80	0.54	0.80	0.85	0.83	0.52	−0.14	0.75	≤0.001
PSME2	proteasome (prosome,macropain) activator subunit 2		Cfa.14895	2.06	2.14	2.45	1.67	0.62	0.14	0.44	0.46	0.35	0.13	−0.15	0.21	≤0.001
PSME2	proteasome (prosome,macropain) activator subunit 2		Cfa.2102	1.11	1.12	1.65	1.45	0.39	0.05	0.29	0.31	0.17	−0.07	−0.31	0.08	≤0.001
SDC2	syndecan 2		Cfa.5258	1.45	1.56	1.05	0.26	0.87	0.38	0.60	0.52	0.65	0.15	0.01	0.08	0.004
SFRS2	splicing factor, arginine/serine-rich 2		Cfa.10605	1.48	1.69	1.59	0.99	0.89	0.85	0.69	0.94	0.71	0.95	0.60	0.79	0.043
SLBP	stem-loop binding protein		Cfa.12597	1.14	1.30	1.12	0.93	0.58	0.47	0.72	0.51	0.34	0.33	−0.34	0.22	0.020
STAT1	signal transducer and activator of transcription 1		Cfa.20754	1.38	1.21	1.78	1.32	0.15	−0.01	0.63	0.16	−0.12	0.08	−0.21	0.09	≤0.001
STAT1	signal transducer and activator of transcription 1		CfaAffx.15419	3.49	3.07	2.74	2.08	0.19	−0.23	0.60	−0.41	−0.70	−0.76	−1.07	−1.11	≤0.001
WARS	tryptophanyl-tRNA synthetase		Cfa.19799	0.50	0.74	1.17	0.77	0.58	0.09	0.20	0.35	0.10	0.23	0.19	0.08	≤0.001
YWHAE	tyr 3-monooxygenase/trp 5-monooxygenase activation protein, epsilon		Cfa.19484	1.53	1.70	1.20	0.55	0.65	0.75	0.56	0.53	0.59	0.19	0.09	0.17	0.006
YWHAE	tyr 3-monooxygenase/trp 5-monooxygenase activation protein, epsilon		Cfa.4461	1.40	1.44	1.18	0.35	0.54	0.76	0.44	0.65	0.64	0.46	0.32	0.46	0.019

a Mean gene expression ratios (log2) for selected genes is relative to the mock-infected ferret gene expression at 2 DPI.

b Statistical significance of gene expression differences over time is determined by EDGE analysis as described in the Methods.

c *Canis familiaris* UniGene Build #11 (April, 2005) identifiers as per the Affymetrix GeneChip Canine Genome 2.0 Array probe library.

The pro-inflammatory cytokine, IL-6 is known to function in various systems such as B cell development, T cell activation and macrophage proliferation [28]. Furthermore, it is a significant regulator of fever during infection. We found that the expression of IL-6 and seven IL-6 signaling-associated genes, including GRB2, JUN, NFKBIA, RRAS2, STAT3, and two MAP kinases (**Table S1**), were increased in ferret lungs as early as 2 DPI and until 7 DPI (**Figure 4C**). These genes were not increased following reinfection or were decreased. IL-6 and STAT3 gene expression was confirmed by QRT-PCR (**Figure S1**). The context of these upregulated molecules at their 5 DPI peak in the IL-6, IL-1, and TNF-α signaling pathways was analyzed by IPA and drawn into the respective pathways (**Figure 5D**).

### Differential IFN Responses in SARS-CoV Infected versus Reinfected Ferrets

**Figure 6:**
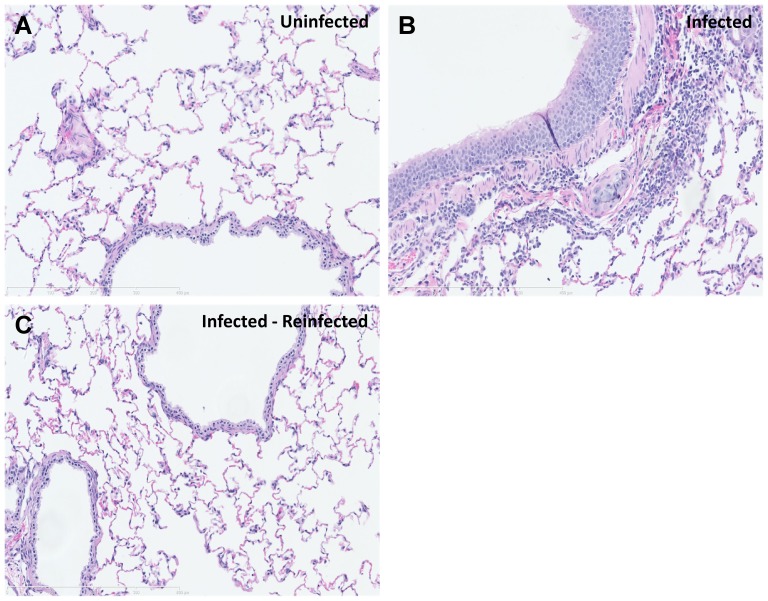
IFN responses in SARS-CoV infected-reinfected ferret lungs. (A) Fifty IRGs selected by pathway analysis are shown in a one-way hierarchical cluster. (B) IPA canonical IFN-signaling pathway analysis at 3 DPI and 3 DPR. All genes are significantly differently expressed (EDGE analysis: ≥2-fold change in at least one time point, p≤0.05, and q≤0.1).

In our previous analysis of host immune correlates with pathogenic potential in SARS-infected individuals, we reported that hyper innate IFN and IFN response gene (IRG) activity could be identified in acutely-infected SARS-CoV patients, the persistence of which correlated with ineffective development of adaptive immunity and severe clinical course (**Table S1**) [5], [9]. Here we found fifty IRGs were significantly upregulated in ferret lung tissue during the first 2–7 DPI with SARS-CoV relative to mock-infected ferrets, including CD274, IFI30, IFI44, IFI44L, IFI6, IRF1, IRF2, ISG15, MNDA, OAS2, and PSMB8 genes (**Table 3** and **Figure 6A**). QRT-PCR validation was performed on CD274, IRF1, IRF2, and MNDA (**Figure S1**).

STAT1 and JAK1, key upstream mediators of integrated IRG gene expression upon phosphorylation, were significantly upregulated in ferret lungs only during acute SARS-CoV infection. STAT1 gene expression was confirmed by QRT-PCR (**Figure S1**).

Two IFN-induced chemokines, CCL2 and CXCL10, were significantly upregulated in the lung at 5–7 DPI with SARS-CoV but not after reinfection (**Table 3** and **Figure 6A**). QRT-PCR validation of CXCL10 gene expression is shown (**Figure S1**).

Interestingly, an additional EDGE analysis of the 3454 genes identified above as significantly differentially expressed across all time points performed using only the 28 DPI through 28 DPR time points identified only 29 significantly increased genes relative to 28 DPI, one of which was an IRG, namely ISG15 (**Table S1**).

The integrated expression of innate immune genes, IRGs, and IFN-mediated chemokines in the lungs of viremic SARS-CoV infected ferrets therefore reflected the antiviral responses that correlate with only the acute phase of SARS.

### Select IRG Expression in Adjuvant-vaccinated SARS-CoV-challenged Ferrets

The ferret also shows promise in the evaluation of candidate SARS vaccines [5], [9], [29]. Past attempts at developing a CoV vaccine have been unsuccessful as many of the candidate vaccines caused disease exacerbation, due to cellular or humoral immune enhancement via antibodies to the outer envelope protein [9], [10]. Above, we have shown an immune profile correlating with primary SARS-CoV infection in ferrets. Furthermore, SARS-CoV reinfection 30 days following primary infection showed a significant decrease in innate and adaptive immune gene activity associated with immune protection. Therefore as a comparison, we next profiled the immune response of SARS-CoV vaccinated ferrets challenged with SARS-CoV to determine if a protection molecular signature could be induced following vaccination.

To investigate the a vaccination molecular signature, ferrets were mock-vaccinated as control or vaccinated using an inactivated whole virus SARS-CoV vaccine candidate formulated with or without Al(OH)_3_, SARS FCP-A and SARS FCP respectively [30]. EDGE microarray analysis was then preformed on lung RNA collected at 2, 7, and 28 days post SARS-CoV challenge (DPC). Of the 3717 significantly altered genes resulting from this combined time course analysis, 42 were identified as IRGs by IPA (**Figure 6A**), many of which had also been identified in the infection-reinfection experiment (**Table 3**). When we applied a two-fold mean difference biological filter between the three groups at each time point, however, it was found that 8 of the 42 IRGs were downregulated at least two-fold at 2 or 7 DPC in ferrets previously immunized with SARS FCP-A relative to the mock-vaccinated group (**Figure 6B**). These 8 IRGs, included: ISG15 (QRT-PCR shown in **Figure S1**), CCL2, IFI44, and PSMB8 discussed above; GPX1 (an antioxidant enzyme), and PSME1 and 2 (roles in MHC class I antigen processing, see **Table 3** for full gene names); and myxovirus resistance 1 (MX1, a potent antiviral). Interestingly, the temporal expression patterns of 4 IRGs (ISG15, IFI44, PSME2 and CCL2) were similar amongst the SARS-CoV infection-reinfection experiments (50 IRGs) and this list of 8 IRGs from the adjuvanted vaccine–challenge experiments (**Figure 6C**). These data show a downregulation of early SARS-CoV challenge regulated ISG genes in the context of a SARS vaccine.

## Discussion

Currently there exists a paucity of information surrounding the molecular events associated with protection from SARS-CoV infection. Furthermore, due to the evolutionary and promiscuous nature of the SARS-CoV and other coronaviruses there is a current need to develop vaccination and therapeutic strategies for humans and mammals. Our time course analysis of differential gene expression in the lungs of SARS-CoV infected and SARS-CoV reinfected ferrets identified three key clusters of functionally related genes. Early (2–14 DPI) expression of IL-6 signaling/complement and IFN response genes followed immediately (14 DPI) by antigen processing and presentation gene expression correlated with peak SARS-CoV titres in the lungs and peak neutralizing antibody titers, respectively. Reinfecting the ferrets with SARS-CoV did not reinitiate the same organized expression of antiviral innate immune response genes; however adaptive immunity in the form of SARS-specific antibody production quickly appeared at 3 DPR. These correlations suggested that a period of IFN-driven innate antiviral responses mediates acute SARS-CoV infection, after which specific adaptive immune responses confer protection. These findings are specifically imperative to the understanding of SARS-CoV and SARS-like-CoV infections and provide a comprehensive baseline for the development of CoV antiviral therapeutics and vaccines tailored to account for the specific IFN responses and subsequent antibody production.

A protective role for complement in SARS-CoV pathogenesis has been proposed [31]; however, the extent to which complement is involved in SARS-CoV infection is unknown. In this study we found that certain complement system genes were significantly upregulated during the first 2 weeks post infection. CFB is key to the alternative complement cascade and is cleaved into Ba and Bb (UNIPROT). The Bb serine protease combines with C3b to generate the C3 convertase. C1q is the first target recognition protein of the classical complement cascade and is an important link between innate and adaptive immunity [32]. C1 complex activity is regulated by C1NH which forms a complex with C1r, C1s, and MASP proteases. We have previously shown that C1NH is upregulated in SARS-CoV infected patients prior to either recovery or progression to severe disease [33]. C4 is cleaved by activated C1 to produce C4b, an essential component of the C3 and C5 convertases of the classical pathway (UNIPROT). FCN1 initiates the lectin complement cascade via MASP zymogens, e.g. MASP1, which in turn cleave C4 [34]. Interestingly, the complement signaling regulators, C1NH and CR1, were also upregulated at 2 DPI.

IL-6 has been shown to be induced in human bronchial epithelial cells following SARS-CoV infection *in vitro* and *in vivo* as well as associated with hyper-immune activation during SARS-CoV infection [14], [35], [36]. We found that the expression of IL-6 and IL-6 signaling-associated genes were increased in ferret lungs for a longer period of time than complement genes (2 DPI to 7 DPI). IL-6-regulated genes are mainly induced by IL-6/IL6 receptor signaling via JAK2:STAT3 and the RAS/MAP/NF-IL6 pathway [37]. IL-6 gene expression is also partially dependent on NF-κB which is activated by NFKBIA (I-κBα) degradation [38]. Since IL-6 signaling can drive further IL-6 expression via the RAS/MAP kinase cascade and NF-IL6 activation [37] our results suggest that potentially self-sustaining acute phase responses occur during early SARS-CoV infection and not after reinfection.

Critical to IFN-mediated antiviral activity is JAK/STAT signaling which prompts widespread transcriptional activation of IRGs [39]. Resembling the results of our PBMC study of acute SARS-CoV infection [33] and the previous results of a SARS-CoV mouse model [14], [40], a prominent number of IRGs were upregulated in ferret lung tissue during the first 2–7 DPI with SARS-CoV infection relative to mock-infected ferrets. CD274 is induced by IFNs and functions in T cell costimulation during viral infection [41]. IFI30 and PSMB8 are induced by IFN-γ and have roles in antigen presentation (UNIPROT). IFI44, IFI44L, IFI6, MNDA, and OAS2 are classical antiviral IRGs induced mainly by type I IFNs (UNIPROT). IRF1 is rapidly induced by IFNs and binds to the upstream regulatory region of type I IFN and IFN-inducible MHC class I genes [42]. IRF2, conversely, binds to the interferon consensus sequence and represses IRF-mediated genes [43].

While we show that IRF/IRG gene expression is integrated during acute infection in our non-fatal ferret SARS-CoV infection model, we previously identified dysregulated IFN activity in SARS-CoV infected patients with severe clinical course [33], suggesting that differently balanced IRG gene signatures affect antiviral versus proinflammatory host immune response development [44]. STAT1 phosphorylation and IRG transcription invokes a cellular antiviral state where STAT1 has been previously reported up-regulated during SARS-CoV infection [13], [45]. STAT1 and JAK1 were indeed significantly upregulated in ferret lungs only during acute SARS-CoV infection. STAT1-deficient mice suffer worse disease upon SARS-CoV infection with greater systemic infection and viral burden in the lungs [13], [46].

Two IFN-induced chemokines, CCL2 and CXCL10, were significantly upregulated in the lung at 5–7 DPI with SARS-CoV infection but not after reinfection. CCL2 protein expression has been associated with early inflammatory host responses during murine SARS-CoV infection [47]. We previously demonstrated that high levels of CXCL10 protein were present in plasma from SARS-CoV infected patients during early illness and quickly returned to baseline levels in those patients that quickly recovered [33], [48]. In severe cases of SARS-CoV infection, however, CXCL10 levels remained significantly elevated for the duration of the patient’s infection. Interestingly, ISG15 was the only known IRG that was significantly increased following reinfection. ISG15 is an antiviral ubiquitin-like modifier that conjugates with JAK1 and STAT1 after type I IFN stimulation [49]. Also, antiviral ISG15 derivatives have been shown to be preferred substrates for the deubiquitinating activity of the SARS-CoV papain-like protease [50]. ISG15 is expressed by a wide variety of lymphoid cells and tissues. ISG15 and its targets may therefore represent unique innate immunity correlates that are not influenced by tissue viral loads, but that may participate in the IFN-mediated transition from innate to adaptive immunity.

Collectively, the marked expression of IFN-mediated responses in the lungs of viremic SARS-CoV infected ferrets and not reinfected ferrets further asserts that proinflammatory IFN responses complement the acute phase of SARS and that resolution of IRG activity is associated with priming of an anamnestic response that will neutralize SARS-CoV reinfection without reinitiating acute inflammation. Although, it is important to mention the lack of upregulation following second infection may be due to the low virus replication levels after secondary infection. The presence of a low immune stimulation in the reinfection group is probably associated with the marginal viral replication. Also, the analysis of the gene expression profiles is suggestive of a scenario with very limited immune stimulation and without prominent participation of suppressor genes.

The general lack of IFN-mediated immune responses in ferrets reinfected with SARS-CoV suggested that a SARS vaccine–challenge experiment would reveal additional immune correlates of immunity. Our microarray analysis on SARS-CoV challenged ferret lungs with or without previous immunization using an Al(OH)_3_-adjuvanted, inactivated whole virus SARS-CoV vaccine candidate revealed the downregulation of 8 IRGs. Moreover, four IRGs (ISG15, IFI44, PSME2 and CCL2) were expressed in common between the SARS-CoV infection-reinfection experiments and these 8 IRGs. The select abrogated expression of IRGs in SARS FCP-A vaccinated, SARS-CoV challenged ferrets reflect the infection-reinfection results demonstrating lack of IRG induction upon reinfection. The expression of these genes early in the natural course of infection suggested IRGs to be indicative of an early stage in SARS-CoV disease, but neither reinfection nor SARS vaccination–challenge provided evidence for IRGs as biomarkers of immune responses that are capable of suppressing SARS-CoV challenge. Importantly, the focus of this study was on the gene expression and protein analysis would be a valuable addition to future experiments. Furthermore, when we measured SARS-CoV viral RNA levels in SARS-CoV challenged mock-, SARS FCP-, and SARS FCP-A-vaccinated ferrets lungs by PCR, all mock-vaccinated ferrets were PCR-positive for SARS-CoV at 7 DPI, one SARS FCP-vaccinated ferret was positive at each of 2 and 7 DPI, and no SARS FCP-A-vaccinated ferrets were PCR-positive for SARS-CoV at any time.

Concerns regarding immune enhancement arose when immunization of ferrets with recombinant modified vaccinia Ankara (rMVA) expressing SARS-CoV spike induced strong inflammation and hepatitis [29], later attributed to rMVA expressing SARS-CoV antigens [9]. More recently, formalin-inactivated whole-virus [24] and adenovirus-based [22], [23] SARS-CoV vaccine candidates have shown promise in reducing pneumonia during SARS-CoV challenge in ferrets. Interestingly, immune enhancement was not seen in mice vaccinated with Al(OH)3-adjuvanted or non-adjuvanted inactivated SARS-CoV whole virus candidate vaccines [30]. Furthermore, vaccine strategies for coronaviruses in an aged mouse model have also been investigated and found that wild type VRP (Venezuelan equine encephalitis virus replicon particle) vaccines protected animals from challenge [15]. Here, IFN-mediated immune enhancement was not noted in our ferret SARS-CoV infection-reinfection model and we have previously reported that increased neutralizing antibody titers after SARS-CoV reinfection is a correlate of immune protection [51]. One caveat, due to the large nature of our study, was the absence of an Alum control only group and it would be important in the future to investigate the genes regulated by Alum treatment alone.

Taken together, this study has given insight into the molecular events following SARS-CoV infection as well as identified an imperative signature for immunological protection against SARS-CoV in both a reinfection and vaccination model. Specifically, the ferret model of SARS-CoV infection showed a two step temporal paradigm of host immune responses in the lung. SARS-CoV infection in the ferrets was characterized by an early innate immune response (robust IFN and chemokine gene expression) followed by adaptive immunity likely eliciting localized anti-SARS-CoV antibodies and protective immune responses. Likewise, the lack of repeat robust innate immune responses in ferrets when reinfected with SARS-CoV suggested that the post-reinfection gene signatures may reveal immune correlates related to successful immunization that may be applied to future use of the ferret model of SARS infection in vaccination strategies. Thus, the identified IRGs have the potential to be inverse correlates of immunity against acute SARS-CoV infection versus reinfection, or SARS-CoV vaccination versus challenge. This dataset serves as a resource in human SARS-CoV vaccine candidate trials for modeling vaccine efficacy in populations at risk for severe outcome.

## Methods

### Ethics Statement

All work with animals was conducted in strict accordance with the Institutional Animal Care and Use Committee (IACUC) guidelines from Southern Research Institute (SRI) or Lovelace Respiratory Research Institute (LLRI). For SRI, ferret studies were approved by the Southern Research Institute’s Institutional Animal Care and Use Committee. Southern Research Institute has Veterinary Medicine tasked to monitor and support all animal experiments. Research was conducted in compliance with the Animal Welfare Act and other federal statutes and regulations relating to animals and experiments involving animals and adheres to principles stated in the Guide for the Care and Use of Laboratory Animals, National Research Council, 1996. The facility where this research will be conducted is fully accredited by the Association for Assessment and Accreditation of Laboratory Animal Care International. For LLRI, The animal work was reviewed and approved by the LRRI Institutional Animal Care and Use Committee under protocol number 06–017. All work involving infectious agents are reviewed by the LRRI Infectious Agents Committee and approved. All infections and sample collections were performed under 5% isofluorane anesthesia and all efforts were made to minimize suffering.

### Cell Culture, Viruses and Vaccine

SARS-CoV TOR2 was amplified in Vero E6 cells (ATCC, VERO C1008, CRL 1586) at the Southern Research Institute BSL-3/ABSL-3 facility as described. The SARS-CoV strain used for vaccine preparation was the CDC strain, AY714217 [30].

### Study Design

Influenza-free, castrated and descented male Fitch ferrets (*Mustela putorius furo*), 36 to 45 weeks of age, were purchased from Triple F Farms (Sayre, PA). Infection-reinfection: 72 ferrets were randomly assigned to groups: (1) mock primary infection (n = 18), (2) primary infection (n = 18), (3) mock infection-reinfection (n = 18), and (4) infection-reinfection (primary and secondary inoculation) (n = 18). On day 0, the group two and four ferrets were anesthetized and received 500 µL of serum-free medium containing SARS-CoV per nare at a total dose of 10^3^ TCID_50_. On 29 DPI, i.e. 0 DPR, group four ferrets were inoculated again with 10^3^ TCID_50_ SARS-CoV. The mock groups received 1 ml of serum-free medium. Three group one and two ferrets were sacrificed at 2, 3, 5, 7, 14, and 28 DPI. Three group three and four ferrets were sacrificed at 2, 3, 5, 7, 14, and 28 DPR. Vaccine–challenge: 57 ferrets were assigned to groups: (1) mock vaccine with SARS-CoV challenge (n = 18), (2) SARS-CoV vaccine with no adjuvant and SARS-CoV challenge (n = 18), (3) SARS vaccine with adjuvant and SARS-CoV challenge (n = 18), and (4) mock vaccine–challenge (n = 3). Vaccinated ferrets received a double-inactivated whole virus SARS-CoV vaccine candidate (Baxter Innovations GmbH, Vienna, Austria, Bulk Drug Substance: SA/O/05/01/R/PBDS) formulated with (SARS FCP-A, 5µg/ferret/dose) or without (SARS FCP, 10 µg/ferret/dose) Al(OH)_3_ as adjuvant [30]. Mock-vaccinated ferrets received TBS+0.05% poloxamer (Baxter, Product Specification #SP0050). Inoculation with 10^3^ TCID_50_ SARS-CoV occurred 42 days post primary vaccination with a boost delivered two weeks prior to infection. Six ferrets from groups one, two, and three were sacrificed at 2, 7, and 28 DPC. Mock vaccine–challenge ferrets were sacrificed at 2 DPC. All procedures were in accordance with the NRC Guide for the Care and Use of Laboratory Animals, the Animal Welfare Act, and the CDC/NIH Biosafety in Microbiological and Biomedical Laboratories. All animal experiments were conducted in a registered BSL-3/ABSL-3 facility at the Lovelace Respiratory Research Institute.

### Viral Neutralization Assay and Quantification

Neutralization tests in serum and TCID_50_ quantification of SARS-CoV in lung tissue suspensions were performed as described. Virus titres were expressed as TCID_50_ units (U)/g. The lower limit of detection was 10^2^ TCID_50_ U/g. RT-PCR detection (within 30 cycles) of SARS-CoV was performed on isolated lung RNA as previously described [52] using ORF1b F:5′-CAGAACGCTGTAGCTTCAAAAATCT-3′, R:5′-TCAGAACCCTGTGATGAATCAACAG-3′ primers.

### Microarray Analysis

Total RNA from lung was prepared for microarray analysis as described, as well as microarray dataset preprocessing and normalization against the appropriate mock dataset [53]. RNA quality from one infection-reinfection group four ferret’s 3 DPR lung was insufficient for arraying. Affymetrix Genechip Canine Genome 2.0 arrays (Affymetrix, Santa Clara, CA) were used to assess ferret-like gene expression as previously established [53], [54]. Infection-reinfection: EDGE [25] was used to identify significantly differentially expressed genes over all time points for groups two and four (2 DPI –28 DPR) using the 2 DPI mocks as baseline. Vaccine–challenge: EDGE was used to identify significantly differentially expressed genes in each of groups one, two, and three using the 2 DPC group 4 datasets as baseline, the results of which were combined. Hierarchical clustering, functional pathway analysis (IPA, Ingenuity Systems, Redwood City, CA), and gene annotation (includes the Universal Protein Resource (UNIPROT, URL: www.expasy.org/uniprot) was performed as previously described [53]. Datasets are available at the NCBI Gene Expression Omnibus, #GSE11704.

### QRT-PCR

QRT-PCR was performed in triplicate as described [53]. Ferret primer sequences: **C1NH**, F:5′-GCCTCTCAGAGCCTGTATGG-3′, R:5′-CTTCCACTTGGCACTCAGGT-3′, **CD274**, F:5′-GGCAATGTGACAATGGAATG-3′, R:5′-CTCTGGCTGTAGCTGCTGTG-3′, **CXCL10**, F:5′-CTTTGAACCAAAGTGCTGTTCTTATC-3′, R:5′-AGCGTGTAGTTCTAGAGAGAGGTACTC-3′, **FCN1**, F:5′-CACCAAGGACCAGGACAATGA-3′, R:5′-CACCAGGCCCCCTGGTA-3′, **IL6,** F:5′-AGTGGCTGAAACACGTAACAATTC-3′, R:5′-ATGGCCCTCAGGCTGAACT-3′, **IRF1**, F:5′-CTACCTGCAGCCTCCACTTC-3′, R:5′-GAGACCAACACGGTCAGGTT-3′, **IRF2**, F:5′-AGGTGACCACCGAGAGTGAC-3′, R:5′-CCCCATGTTGCTGAGGTACT-3′, **ISG15**, F:5′-AGCAGCAGATAGCCCTGAAA-3′, R:5′-CAGTTCTTCACCACCAGCAG-3′, **MNDA**, F:5′-CCTGCGTGGACAAGCTAATA-3′, R:5′-GCTGCTTCTTCTTGGGAGTT-3′, **STAT1**, F:5′-AGCCTTGCATGCCAACTCA-3′, R:5′-ACAGTCCAGCTTCACCGTGAA-3′, and **STAT3**, F:5′-CAACCCCAAGAACGTGAACT-3′, R:5′-AGCCCACGTAATCTGACACC-3′.

### Histopathology

On Day 7 pI, animals were euthanized and the upper and lower left lobes of the lung tissues were harvested and formalin-fixed by perfusion followed by paraffin embedding and sectioning. Tissue slides were then stained with hematoxylin and eosin for histopathology assessment.

### Statistical Analysis

The microarray analysis used EDGE time course modeling [54] with p≤0.05 and q≤0.1 for significance and false discovery rate, respectively, and ≥2-fold change in at least one time point.

## Supporting Information

Figure S1 **QRT-PCR Validation of microarray analysis of ferret lungs infected and reinfected with SARS-CoV or vaccinated and SARS-CoV infected.** QRT-PCR analysis of gene expression in lung tissue from ferrets infected and reinfected with SARS-CoV (A-J). QRT-PCR analysis of ISG15 expression in lung tissue from SARS vaccinated–challenged ferrets. Values shown represent the groups’ mean mRNA levels for the indicated time points relative to the mean mRNA levels for the appropriate mock animals at 2 DPI (A-J) or 2 DPC (K). All analyses were performed in triplicate. Group means represent data from 3 ferrets. DPI, days post-infection. DPR, days post-reinfection. DPC, days post-challenge. Error bars represent standard error. * p = 0.002 (Student’s t-test) difference from the mock, ** non-significant difference from the mock.
(TIF)

Table S1 **Genes significantly changed over time in SARS-CoV infected ferret lungs following reinfection.**
(DOC)
