# Supplementary figures and images for: Lack of Innate Interferon Responses during SARS Coronavirus Infection in a Vaccination and Reinfection Ferret Model
Source: PLoS One. 2012 Sep 24;7(9):e45842. doi: 10.1371/journal.pone.0045842 (PMC3454321; doi:10.1371/journal.pone.0045842)

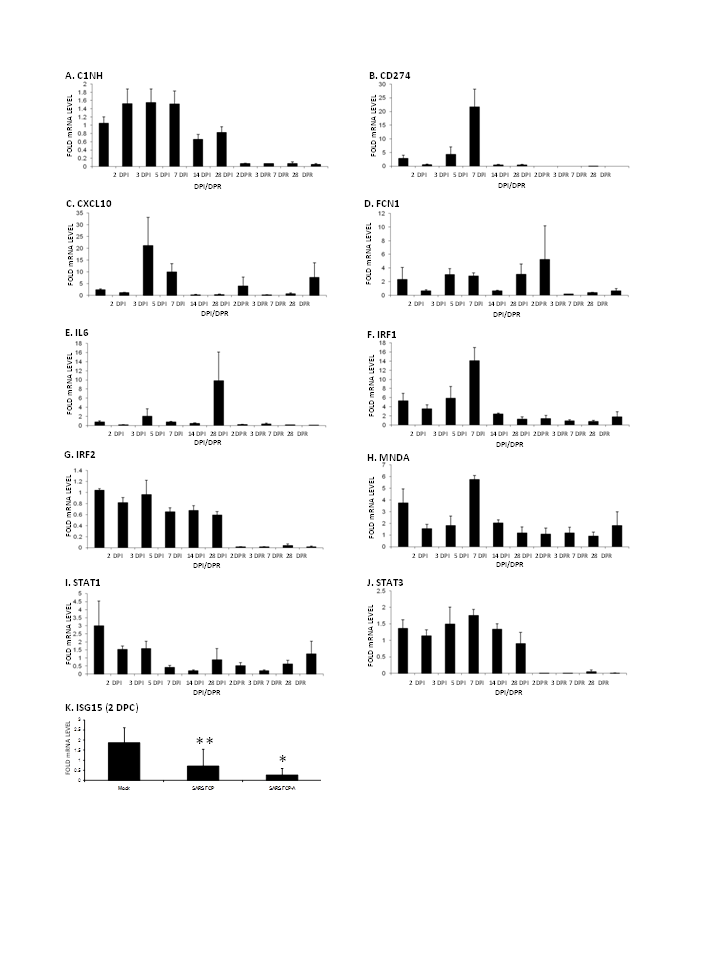

Supplement: Figure S1 — QRT-PCR Validation of microarray analysis of ferret lungs infected and reinfected with SARS-CoV or vaccinated and SARS-CoV infected. QRT-PCR analysis of gene expression in lung tissue from ferrets infected and reinfected with SARS-CoV (A-J). QRT-PCR analysis of ISG15 expression in lung tissue from SARS vaccinated–challenged ferrets. Values shown represent the groups’ mean mRNA levels for the indicated time points relative to the mean mRNA levels for the appropriate mock animals at 2 DPI (A-J) or 2 DPC (K). All analyses were performed in triplicate. Group means represent data from 3 ferrets. DPI, days post-infection. DPR, days post-reinfection. DPC, days post-challenge. Error bars represent standard error. * p = 0.002 (Student’s t-test) difference from the mock, ** non-significant difference from the mock. (TIF) [file pone.0045842.s001.tif]
