# Supplementary material for: Lack of Innate Interferon Responses during SARS Coronavirus Infection in a Vaccination and Reinfection Ferret Model
Source: PLoS One. 2012 Sep 24;7(9):e45842. doi: 10.1371/journal.pone.0045842 (PMC3454321; doi:10.1371/journal.pone.0045842)
Supplement: Table S1 — Genes significantly changed over time in SARS-CoV infected ferret lungs following reinfection. (DOC) [file pone.0045842.s002.doc]

**Table S1. Genes significantly changed over time in SARS-CoV** infected ferret lungs following reinfection.

|  |  |  | Gene Expression (Days Post Reinfection)^a^ | | | | | |  |
| --- | --- | --- | --- | --- | --- | --- | --- | --- | --- |
| Gene | Gene Name | Ferret-like  Gene ID^c^ |  |  |  |  |  |  | EDGE  p value^b^ |
|  |  |  | 2 | 3 | 5 | 7 | 14 | 28 |  |
| ANKRD11 | ankyrin repeat domain 11 | Cfa.5830 | 0.176 | 0.438 | 0.353 | 1.027 | 0.984 | 1.134 | ≤0.001 |
| B3GALT2 | 3beta-galactosyltransferase 2 | Cfa.3036 | -0.414 | -0.348 | -0.009 | 0.447 | 0.624 | 1.414 | 0.009 |
| CCDC34 | coiled-coil domain containing 34 | Cfa.18019 | 0.016 | -0.031 | 0.393 | 0.707 | 1.290 | 1.050 | ≤0.001 |
| CUTL2 | cut-like homeobox 2 | CfaAffx.7644 | -0.084 | 0.134 | 0.359 | 0.959 | 0.973 | 1.288 | ≤0.001 |
| DAPK1 | death-associated protein kinase 1 | CfaAffx.2872 | 0.704 | 0.848 | 0.279 | 2.096 | 1.818 | 1.714 | ≤0.001 |
| DNAJC21 | DnaJ (Hsp40) homolog, subfamily C, member 21 | CfaAffx.28709 | 0.627 | 0.717 | 0.369 | 1.086 | 0.860 | 0.505 | 0.006 |
| FAM73B | family with sequence similarity 73, member B | CfaAffx.30620 | -0.029 | 0.047 | 0.008 | 1.119 | 0.634 | 1.010 | 0.012 |
| GK | glycerol kinase | Cfa.19596 | 0.322 | 0.055 | 0.066 | 0.513 | 0.608 | 1.280 | ≤0.001 |
| ISG15 | ISG15 ubiquitin-like modifier | Cfa.10757 | 1.152 | 0.382 | 0.337 | -0.313 | -0.150 | -0.080 | 0.035 |
| LGALS8 | lectin, galactoside-binding, soluble, 8 | Cfa.11075 | 0.254 | 0.539 | 0.137 | 1.061 | 0.736 | 1.127 | 0.008 |
| NKX2-5 | NK2 transcription factor related, locus 5 | CfaAffx.25653 | -0.852 | -0.590 | 0.117 | 0.581 | 1.588 | 0.741 | 0.005 |
| PDK4 | pyruvate dehydrogenase kinase, isozyme 4 | Cfa.2282 | 0.804 | 0.692 | 0.735 | 0.863 | 0.987 | 2.169 | 0.013 |
| PPP1R12A | protein phosphatase 1, regulatory subunit 12A | CfaAffx.9639 | 0.288 | 0.286 | 0.354 | 0.754 | 1.290 | 1.084 | ≤0.001 |
| PRPF40A | PRP40 pre-mRNA processing factor 40 homolog A | CfaAffx.9716 | 0.13 | 0.183 | 0.167 | 0.766 | 0.954 | 1.071 | ≤0.001 |
| RFC1 | replication factor C (activator 1) 1, 145kDa | Cfa.19342 | 0.2 | 0.344 | 0.367 | 1.214 | 1.287 | 1.293 | ≤0.001 |
| SFRS18 | splicing factor, arginine/serine-rich 18 | Cfa.15354 | -0.155 | -0.022 | 0.005 | 0.986 | 1.216 | 1.275 | ≤0.001 |
| SGMS1 | sphingomyelin synthase 1 | CfaAffx.23960 | 0.085 | 0.223 | 0.139 | 0.726 | 0.562 | 1.029 | 0.007 |
| SNRPD1 | small nuclear ribonucleoprotein D1 | Cfa.6238 | 0.311 | 0.412 | 0.367 | 0.997 | 0.796 | 1.070 | 0.002 |
| SON | SON DNA binding protein | Cfa.11296 | -0.006 | 0.286 | 0.124 | 1.116 | 1.320 | 1.129 | ≤0.001 |
| STAG1 | stromal antigen 1 | CfaAffx.12000 | -0.064 | 0.036 | 0.105 | 0.618 | 0.531 | 1.043 | ≤0.001 |
| SUB1 | SUB1 homolog (S. cerevisiae) | CfaAffx.25557 | -0.067 | 0.109 | 0.103 | 1.016 | 0.819 | 1.098 | ≤0.001 |
| TAOK3 | TAO kinase 3 | Cfa.20509 | -0.098 | 0.068 | 0.044 | 0.620 | 0.634 | 1.199 | ≤0.001 |
| TAX1BP1 | Tax1 binding protein 1 | CfaAffx.5437 | -0.008 | 0.322 | -0.326 | 1.339 | 0.894 | 1.410 | 0.001 |
| THOC2 | THO complex 2 | CfaAffx.28390 | 0.311 | 0.230 | 0.182 | 0.903 | 1.135 | 0.840 | ≤0.001 |
| TMED5 | transmembrane emp24 protein transport domain containing 5 | Cfa.17716 | 0.173 | 0.020 | 0.292 | 0.680 | 0.720 | 1.040 | ≤0.001 |
| TMEM206 | transmembrane protein 206 | CfaAffx.19138 | 0.42 | 0.621 | 0.260 | 1.073 | 0.924 | 1.041 | 0.008 |
| WDR45L | WDR45-like | Cfa.19109 | 0.129 | 0.176 | 0.425 | 1.370 | 1.153 | 1.531 | 0.036 |
| ZNF292 | zinc finger protein 292 | CfaAffx.5519 | -0.115 | 0.037 | 0.090 | 0.940 | 0.984 | 1.061 | ≤0.001 |
| ZWINT | ZW10 interactor | CfaAffx.23812 | -0.999 | -0.690 | -0.027 | 0.516 | 1.339 | 0.878 | 0.002 |

^a^ Mean gene expression ratios (log2) for selected genes is relative to SARS-CoV infected ferret lung gene expression at 28 DPI.

^b^ Statistical significance of gene expression differences over time is determined by EDGE analysis as described in the Methods.

^c^ *Canis familiaris* UniGene Build #11 (April, 2005) identifiers as per the Affymetrix GeneChip Canine Genome 2.0 Array probe library.
